# Jian-Gan-Xiao-Zhi Decoction Alleviates Inflammatory Response in Nonalcoholic Fatty Liver Disease Model Rats through Modulating Gut Microbiota

**DOI:** 10.1155/2021/5522755

**Published:** 2021-03-20

**Authors:** Jiabao Liao, Xuehua Xie, Jinmei Gao, Zhaiyi Zhang, Fei Qu, Huantian Cui, Yongjun Cao, Xue Han, Jie Zhao, Weibo Wen, Hongwu Wang

**Affiliations:** ^1^Jiaxing Hospital of Traditional Chinese Medicine, Zhejiang Chinese Medical University, Jiaxing, Zhejiang, China; ^2^Nanjing University of Traditional Chinese Medicine, Nanjing, Jiangsu, China; ^3^Yunnan Provincial Hospital of Chinese Medicine, Kunming, Yunnan, China; ^4^Fujian People's Hospital of Traditional Chinese Medicine, Fuzhou, Fujian, China; ^5^Tianjin University of Traditional Chinese Medicine, Tianjin, China; ^6^Shandong Provincial Key Laboratory of Animal Cell and Developmental Biology, School of Life Sciences, Shandong University, Qingdao, China; ^7^Nantong Hospital of Traditional Chinese Medicine, Nantong, Jiangsu, China

## Abstract

**Background:**

Jian-Gan-Xiao-Zhi decoction (JGXZ), composed of *Salvia miltiorrhiza Bunge*, *Panax notoginseng*, *Curcuma zedoaria*, and other 9 types of herbs, has demonstrated beneficial effects on nonalcoholic fatty liver disease (NAFLD). However, the mechanisms behind JGXZ's impact on NAFLD remain unknown.

**Methods:**

In this study, a NAFLD rat model induced by a high-fat diet (HFD) received oral treatment of JGXZ (8 or 16 g crude herb/kg) for 12 weeks. The therapeutic effects of JGXZ on NAFLD model rats were investigated through blood lipid levels and pathological liver changes. 16S rRNA analysis was used to study the changes in gut microbiota after JGXZ treatment. The expressions of occludin and tight junction protein 1 (ZO-1) in the colon were investigated using immunostaining to study the effects of JGXZ on gut permeability. The anti-inflammatory effects of JGXZ were also studied through measuring the levels of IL-1*β*, IL-6, and TNF-*α* in the serum and liver.

**Results:**

JGXZ treatment could decrease body weight and ameliorate dyslipidemia in NAFLD model rats. H&E and Oil Red O staining indicated that JGXZ reduced steatosis and infiltration of inflammatory cells in the liver. 16S rRNA analysis showed that JGXZ impacted the diversity of gut microbiota, decreasing the *Firmicutes*–to-*Bacteroidetes* ratio, and increasing the relative abundance of probiotics, such as *Alloprevotella*, *Lactobacillus*, and *Turicibacter*. Gut permeability evaluation found that the expressions of ZO-1 and occludin in the colon were increased after JGXZ treatment. Moreover, JGXZ treatment could decrease the levels of IL-1*β*, IL-6, and TNF-*α* in the serum and liver.

**Conclusions:**

Our study illustrated that JGXZ could ameliorate NAFLD through modulating gut microbiota, decreasing gut permeability, and alleviating inflammatory response.

## 1. Background

Nonalcoholic fatty liver disease (NAFLD) is a chronic disease characterized by hepatic steatosis and dyslipidemia that can lead to type 2 diabetes, atherosclerosis, hepatic fibrosis, and even hepatic carcinoma [1]. With lifestyles and diets changing for the worse in recent years, the morbidity of NAFLD is on the rise [2]. Currently, few therapeutic approaches have demonstrated a definite effect on NAFLD [3]. As such, there is a critical need to develop novel therapies to treat NAFLD.

Traditional Chinese medicine has been widely used in the treatment of NAFLD, with accumulating numbers of studies highlighting its lipid-lowering effects [4]. For instance, researchers were able to utilize Jiang-Zhi-Ning to ameliorate high-fat-diet (HFD)–induced dyslipidemia through modulating the synthesis and translation of cholesterol and inhibiting oxidative stress [5]. Additionally, Zhixiong capsules have shown lipid-lowering effects on atherosclerosis model rats [6]; and Da-Huang-Ze-Xie decoction can improve dyslipidemia and hepatic steatosis in NAFLD rats through modulating gut microbiota and inhibiting the inflammatory response in the liver [7].

Jian-Gan-Xiao-Zhi decoction (JGXZ), composed of *Salvia miltiorrhiza* Bunge, *Panax notoginseng*, *Curcuma zedoaria*, *hawthorn*, *Astragalus membranaceus*, *Vatica mangachapoi* Blanco, *Radix Paeoniae Rubra*, *Curcuma longa*, *Rhizoma Alismatis*, *Dendranthema morifolium*, *lotus leaf*, and *Glycyrrhiza uralensis* Fisch., has been used for the treatment of NAFLD in clinics. Furthermore, our previous study demonstrated that JGXZ could improve dyslipidemia and insulin resistance in a NAFLD rat model [8]. However, the mechanisms behind JGXZ's beneficial impact on NAFLD remain elusive.

Part of the difficulty is that the mechanisms of pathogenesis for NAFLD itself remain unclear. An imbalance between energy consumption and intake, inflammatory responses in the liver, and hereditary factors have all been implicated in NAFLD [9]. Recent studies have also indicated a critical role for gut microbiota in the progression of NAFLD, with the diversity of gut microbiota altered significantly in NAFLD patients [10, 11]. Studies have also demonstrated that the dysfunction of the gut microbiota could impair the tight junction of intestinal epithelial cells and cause an increase in gut permeability [12]; whereupon metabolites derived from gut microbiota, such as lipopolysaccharide (LPS), could enter circulation through the impaired intestinal mucosal barrier and trigger inflammatory responses in the liver [13]. Modulating gut microbiota to improve gut permeability and reduce inflammatory response has been proposed as a potential method to alleviate NAFLD [14].

Based on the dysfunction in gut microbiota and the inflammatory response in the liver about the potential pathogenesis of NAFLD, as well as our previous results regarding the improvement in dyslipidemia and insulin resistance of JGXZ, our present study was aimed to illustrate the regulatory effects of JGXZ on inflammatory response and gut permeability and correlated gut microbiota in a rat model. An NAFLD rat model was induced via HFD and orally treated with JGXZ (8 or 16 g crude herb/kg) for 12 weeks. The therapeutic effects of JGXZ on NAFLD model rats were investigated through blood lipid levels and pathological liver changes. 16S rRNA analysis was used to study the changes in gut microbiota after JGXZ treatment. Gut permeability was investigated via occludin and tight junction protein 1 (ZO-1) colon immunostaining. Lastly, the anti-inflammatory effects of JGXZ were examined by quantification of IL-1*β*, IL-6, and TNF-*α* in rat serum and livers.

## 2. Material and Methods

### 2.1. Reagents

HFD (17.7% sucrose, 17.7% fructose, 19.4% protein, and 40% fat) was purchased from Beijing Huafukang Bioscience Co., Ltd. (Beijing, China). Total DNA and RNA extraction kits (cat. no. DP419), first-stand cDNA reverse transcription kits (cat. no. KR106-02), polymerase chain reaction (PCR) kits (cat. no. FP205-02), and primers were obtained from Tiangen Biotechnology Co., Ltd. (Beijing, China). Rat IL-6 (cat. no. E02I0006), IL-1*β* (cat. no. E02I0010), and TNF-*α* (cat. no. E02T0008) ELISA kits were obtained from Shanghai BlueGene Biotech CO., Ltd. (Shanghai, China). Aspartate aminotransferase (AST; cat. no. C010-2-1), alanine aminotransferase (ALT; cat. no. C009-2-1), triglyceride (TG; cat. no. A110-1-1), and total cholesterol (TC; cat. no. A111-1-1) test kits were purchased from Nanjing Jiancheng Bioengineering Institute (Nanjing, China). The Oil Red O staining kit (cat. no. G1261) was obtained from Solarbio Biotechnology Co., Ltd. (Beijing, China). Primary antibodies of ZO-1 (cat. no. 61-7300) and occludin (cat. no. 71-1500) were purchased from Invitrogen (USA).

### 2.2. Animals

Male Sprague-Dawley rats (180–220 g) were purchased from Beijing Huafukang Bioscience Co., Ltd. All animals were handled using experimental protocols outlined by the National Institutes of Health regulations and approved by the Ethics Committee and Use Committee of the Yunnan University of Traditional Chinese Medicine (approval no. 2020-0016). Throughout the acclimatization and study periods, all animals had access to food and water *ad libitum* and were maintained on a 12-h light/dark cycle (21 ± 2°C with a relative humidity of 45 ± 10%).

### 2.3. Preparation of JGXZ

JGXZ contained 15 g of *Salvia miltiorrhiza* Bunge, 6 g of *Panax notoginseng*, 15 g of *Curcuma zedoaria*, 20 g of *hawthorn*, 20 g of *Astragalus membranaceus*, 10 g of *Vatica mangachapoi* Blanco, 20 g of *Radix Paeoniae Rubra*, 12 g of *Curcuma longa*, 15 g of *Rhizoma Alismatis*, 15 g of *Dendranthema morifolium*, 15 g of *lotus leaf*, and 6 g of *Glycyrrhiza uralensis* Fisch. All herbs were purchased from the Department of Pharmacy of Yunnan Provincial Hospital of Traditional Chinese Medicine. The above herbs were soaked in 300 mL of water for 30 min and decocted for 30 min to obtain a JGXZ extract. The water extract of JGXZ was then filtered and concentrated to a density of 4 g crude herb/mL.

### 2.4. Animal Grouping

After acclimatization to laboratory conditions for 1 week, 40 rats were weight-matched and randomized into four groups (*n* = 10 per group): control, model, JGXZ low-dose, and JGXZ high-dose groups. Rats in the control group received standard chow containing 59.4% total carbohydrate, 20% protein, and 4.8% fat. Rats in the model, JGXZ low-dose, and JGXZ high-dose groups received HFD for 12 weeks to induce NAFLD [15]. Rats in the JGXZ low-dose and JGXZ high-dose groups received an oral gavage of JGXZ (8 or 16 g crude herb/kg rat weight, respectively) [8], whereas rats in the control and model groups received an oral treatment of 2 mL saline once per day for 12 weeks. Rats in each group were weighed every two weeks.

**Figure 1:**
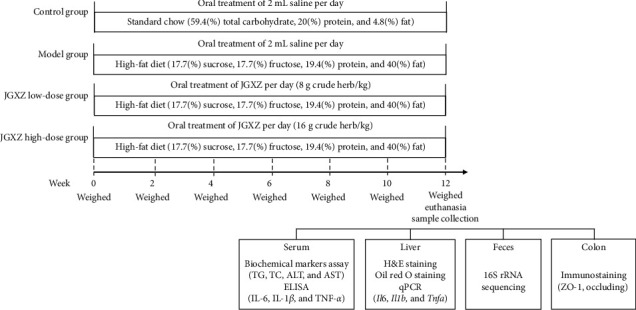
The timeline for experimental design.

At the end of 12 weeks of JGXZ treatment, rat livers were removed and weighed under anesthesia. The liver index was calculated using the following formula: liver index (%) = liver weight (g)/body weight (g) × 100. The timeline for experimental design is shown in Figure 1.

### 2.5. Serum Biochemical Markers Assay

After 12 weeks of JGXZ treatment, serum samples were collected for the biochemical assays. Briefly, rats were anaesthetized and blood was harvested by syringe from the aorta abdominalis. Then, blood was centrifuged at 3,000 rpm for 15 min to obtain the serum. The levels of TG, TC, ALT, and AST in the serum were assayed according to the manufacturer's instructions provided by Nanjing Jiancheng Biological Engineering Institute (Nanjing, China), and the absorbance value was detected using a microplate reader (Varioskan Flash; Thermo Fisher, Massachusetts, USA).

### 2.6. Histology

After 12 weeks of JGXZ treatment, rat livers were removed, fixed in paraffin, and cut into 5 *μ*m sections. Hematoxylin and eosin (H&E) staining was performed using standard protocols. Briefly, after dewaxing, rehydration, staining, dehydration, transparency, and sealing, the pathological changes were visualized under a light microscope (BX50; Olympus America, Melville, NY, USA).

### 2.7. Oil Red O Staining

Oil Red O staining on liver were based on Cui et al. 2020 [16]. Briefly, rat livers were embedded in Tissue-Tek OCT compound (Sakura Finetek) for frozen block preparation. Frozen tissue sections were stained with Oil Red O for lipid detection following the manufacturer's instructions. The staining of lipid drops by Oil Red O was quantified using Image J to obtain the integrated optical density (IOD). The mean optical density (MOD) was calculated based on the ratio of IOD to the sum area.

### 2.8. Fecal 16S rRNA Sequencing

After 12 weeks of JGXZ treatment, feces from the control, model, JGXZ low-dose, and JGXZ high-dose groups were simultaneously obtained under sterile conditions in a laminar flow hood. 16S rRNA sequencing was conducted as described previously [17]. Briefly, total DNAs were extracted from fecal samples using the CTAB/SDS method. DNA purity and quantity were evaluated on 1% agarose gels and subsequently diluted to 1 ng/*µ*L with sterile water. The PCR was carried out with diluted template DNA using specific barcoded primers (515F : GTGCCAGCMGCCGCGGTAA 806R : GGACTACHVGGGTWTCTAAT). The PCR products were visualized after electrophoresis and purified with the GeneJET™ Gel Extraction Kit (Qiagen, Germany). Sequencing libraries were generated using TruSeq® DNA PCR-Free Sample Preparation Kit (Illumina, United States). After library detection, the IlluminaHiSeq2500 platform was used for sequencing. Then, paired-end reads were generated from 16S rRNA sequencing and assigned to samples, truncated using trimming the barcode and primer sequence, and merged based on FLASH V1.2.7 analysis tool (a) to derive raw tags, which were subsequently rarified to obtain the clean tags according to the QIIME V1.9.1 quality controlled process (b). The effective tags were obtained through detecting and removing the chimera sequences in clean tags using the UCHIME algorithm (c). The sequences of effective tags with ≥97% similarity were assigned to the same OTUs via Uparse V7.0.1001 software (d). Then representative sequences for each OTU were selected for further annotation using the SILVA database (e). The relative abundances of OTUs were normalized using a standard of sequence number corresponding to the sample with the least sequences. Ultimately, the normalized data were applied for alpha diversity and beta diversity analysis.

- http://ccb.jhu.edu/software/FLASH/
- http://qiime.org/scripts/split_libraries_fastq.html
- http://www.drive5.com/usearch/manual/uchime_algo.html
- http://drive5.com/uparse/
- http://www.arb-silva.de/

### 2.9. Cytokine Quantification by Enzyme-Linked Immunoassay (ELISA)

The levels of IL-6, IL-1*β*, and TNF-*α* in the serum were measured by ELISA according to the manufacturer's instructions (Shanghai BlueGene Biotech Co., Ltd. China).

### 2.10. RNA Isolation and Real-Time Reverse Transcription Quantitative Polymerase Chain Reaction (qPCR)

We followed the methods of Wang et al. 2020 [18]. Total RNAs were isolated from livers using the RNA extraction kit, and first-strand cDNAs were synthesized from 1 *μ*g of total RNAs according to the manufacturer's instructions. qPCR was used to detect the expression of *Il6*, *Il1b*, and *Tnfa* in the livers. All samples were run in triplicate and detected using a BIORAd iQ5 detection system. *Actb* was used as the loading control. Quantification was done using the 2^−-△△CT^ method [19]. The sequences of all primers are listed in Table 1.

### 2.11. Immunostaining

We followed the methods of Wang et al. 2020 [18]. Briefly, rat colons were removed and fixed in paraffin, and the expression of occludin and tight junction protein-1 (ZO-1) in the colon was accessed via immunostaining. The ratio of positive expressed area to sum area was analyzed and quantified using Image *J* based on the IOD.

### 2.12. Statistics

All data are reported as the mean ± standard deviation (mean ± SD) for independent experiments. Statistical differences between the experimental groups were examined by analysis of variance (ANOVA) using SPSS, version 20.0. A *P* value < 0.05 was considered statistically significant. Curve fitting was carried out using the graphical package GraphPad Prism5.

## 3. Results

### 3.1. Effects of JGXZ on Body Weight Gain, Dyslipidemia, and Liver Pathology in NAFLD Model Rats

**Figure 2:**
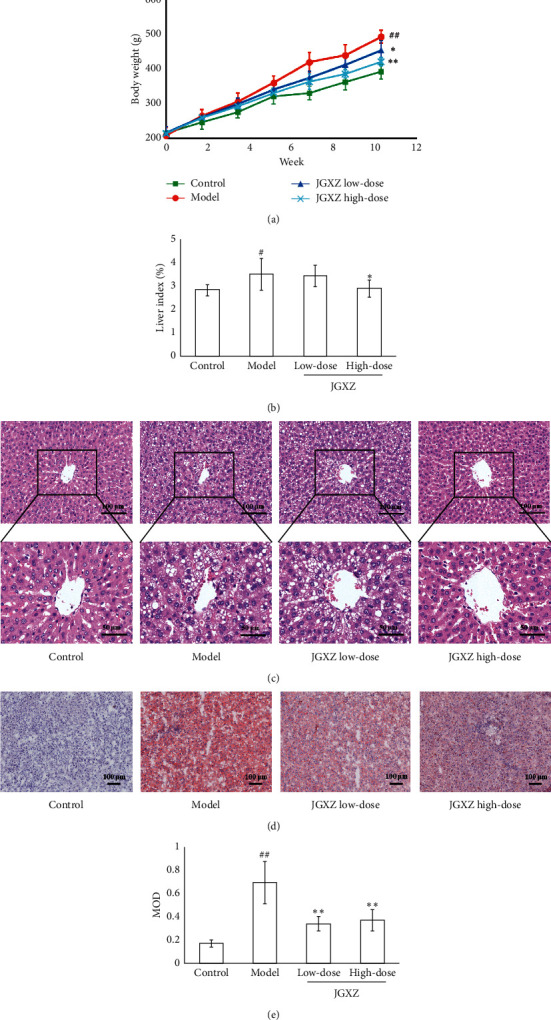
JGXZ treatment reduced body weight gain and improved liver steatosis in NAFLD model rats. (a) Body weight gain was reduced in NAFLD model rats after treatment with JGXZ. (b) JGXZ treatment decreased the liver index in NAFLD model rats. (c) H&E staining indicated that JGXZ treatment ameliorated liver steatosis in NAFLD model rats (200 × and 400 ×). (d, e) The liver lipid contents were decreased in NAFLD model rats after JGXZ treatment (100 ×). Control, model, JGXZ low-dose, and JGXZ high-dose groups (*n* = 10 per group). Data are presented as the mean ± SD. ^#^*P* < 0.05 compared to the control group; ^##^*P* < 0.01 compared to the control group; ^*∗*^*P* < 0.05 compared to the experimental model group; and ^∗∗^*P* < 0.01 compared to the experimental model group.

During the 12weeks of HFD treatment, the body weight in the model group increased significantly compared with the control group (*P* < 0.01). Both low-dose and high-dose JGXZ treatment inhibited the body weight gain in HFD-treated rats (*P* < 0.05 and *P* < 0.01, respectively; Figure 2(a)). After JGXZ treatment for 12 weeks, the liver index was significantly higher in the model group than the control group (*P* < 0.05), whereas the liver index was significantly decreased in the JGXZ high-dose group compared with the model group (*P* < 0.05, Figure 2(b)). Compared with the control group, the serum levels of ALT, AST, TG, and TC were significantly increased in the model group (*P* < 0.01). Low-dose JGXZ-treated rats exhibited significantly lower serum ALT and TG compared with rats in the model group (*P* < 0.05). Accordingly, the serum levels of ALT, AST, TG, and TC were significantly lower in the JGXZ high-dose group compared with the model group (*P* < 0.01, *P* < 0.01, *P* < 0.01, and *P* < 0.05, respectively; Table 2). *H&E* staining indicated extensive steatosis of hepatocytes in the model group, whereas JGXZ treatment alleviated hepatocyte steatosis in NAFLD model rats (Figure 2(c)). Likewise, Oil Red O staining showed increased lipid contents in the model group compared with the control group (*P* < 0.01, Figures 2(d), 2(e)), JGXZ (8 and 16 g crude herb/kg) treatment decreased the lipid contents in the liver (*P* < 0.01; Figure 2(d), 2(e)).

### 3.2. Effects of JGXZ on Gut Dysbiosis in NAFLD Model Rats

**Figure 3:**
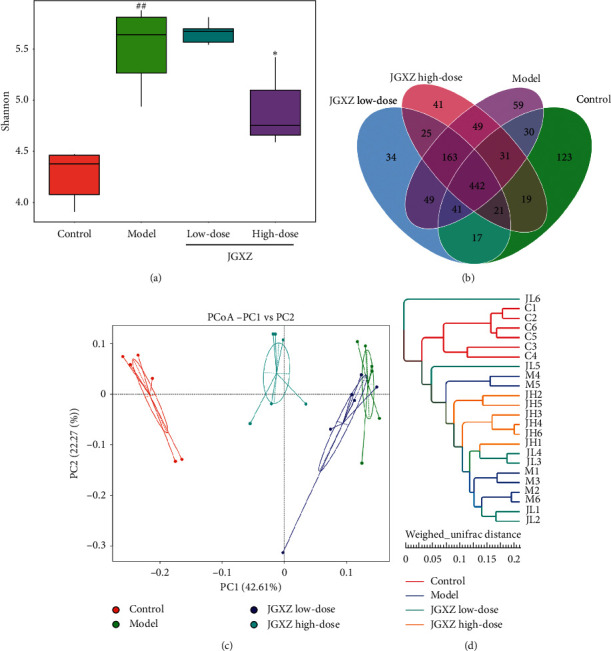
JGXZ treatment improved the diversity of gut microbiota in NAFLD model rats. (a) The Shannon index was decreased in NAFLD model rats after JGXZ treatment. (b) The different numbers of OTUs were visualized via a Venn diagram. (c), (d) PCoA and system clustering tree showed more similar beta diversity between the JGXZ high-dose and control groups than that between the model and control groups. Control, model, JGXZ low-dose, and JGXZ high-dose groups (*n* = 6 per group). ^##^*P* < 0.01 compared to the control group and ^*∗*^*P* < 0.05 compared to the experimental model group.

We next examined whether JGXZ could improve the dysbiosis of gut microbiota using 16S rRNA sequencing. The Shannon index was calculated to determine the alpha diversity of gut microbiota in each group. The Shannon index was higher in the model group compared with the control (*P* < 0.01) and was lower in JGXZ high-dose group compared with the model group (*P* < 0.05, Figure 3(a)). Venn diagram analysis indicated that there were 442 OTUs overlapped among the groups: 544 OTUs present in the control and model groups; 685 in the model and JGXZ low-dose groups; 695 in the model and JGXZ high-dose groups; and 651 in JGXZ low-dose and JGXZ high-dose groups (Figure 3(b)). The beta diversity of gut microbiota was also studied using principle coordinate analysis (PCoA) and system clustering tree. PCoA and system clustering tree indicated a significant variance of beta diversity between the control and model groups. Both low-dose and high-dose JGXZ treatment changed the beta diversity in NAFLD model rats, with the distances between JGXZ-treated groups (both 8 and 16 g crude herb/kg) and the model group in PCA shorter than that between the model and control groups. Additionally, the distances between the high-dose JGXZ-treated rats and the control group showed more similar beta diversities than that between the control group and low-dose JGXZ-treated rats (Figures 3(c), 3(d)).

**Figure 4:**
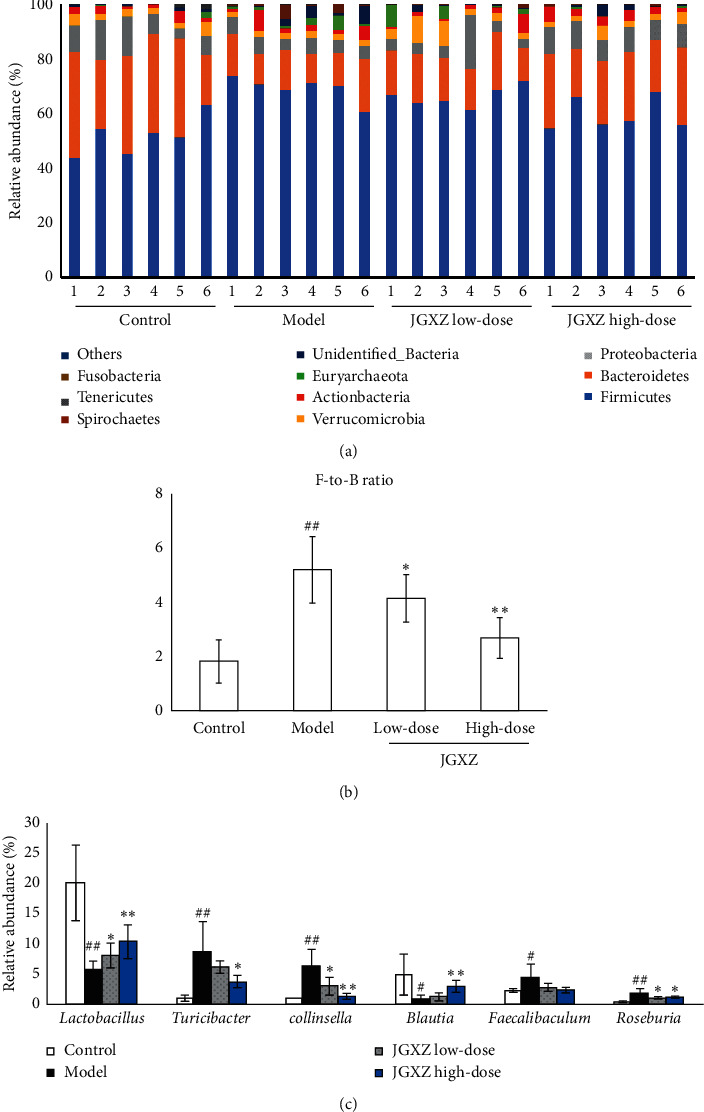
JGXZ treatment altered the abundances of gut microbiota in NAFLD model rats. (a, b) At the phylum level, JGXZ treatment decreased the *F*-to-*B* ratio in NAFLD model rats. (c) JGXZ treatment increased the abundances of *Lactobacillus* and *Blautia* and decreased the abundances of *Turicibacter*, *Collinsella*, and *Roseburia* in the gut. Control, model, JGXZ low-dose, and JGXZ high-dose groups (*n* = 6 per group). ^#^*P* < 0.05 compared to the control group; ^##^*P* < 0.01 compared to the control group; *∗P* < 0.05 compared to the experimental model group; and ^∗∗^*P* < 0.01 as compared to the experimental model group.

We then investigated the changes in abundances of gut microbiota in each group. At the phylum level, *Firmicutes* and *Bacteroidetes* were the most abundant phyla in all samples (Figure 4(a)). The *Firmicutes*-to-*Bacteroidetes* (*F*-to-*B*) ratio was higher in the model group than that in the control group (*P* < 0.01), whereas the *F*-to-*B* ratio was lower in the JGXZ-treated groups (8 and 16 g crude herb/kg) than that in the model group (*P* < 0.05 and *P* < 0.01, respectively; Figure 4(b)). At the genus level, the abundances of *Lactobacillus* (*P* < 0.01) and *Blautia* (*P* < 0.05) were decreased and the abundances of *Turicibacter* (*P* < 0.01)*, Collinsella* (*P* < 0.01)*, Faecalibaculum* (*P* < 0.05), and *Roseburia* (*P* < 0.01) were increased in the model group compared with the control group (Figure 4(c)). Low-dose JGXZ treatment increased the abundance of *Lactobacillus* (*P* < 0.05) and decreased the abundances of *Collinsella* (*P* < 0.05) and *Roseburia* (*P* < 0.05). High-dose JGXZ treatment increased the abundances of *Lactobacillus* (*P* < 0.01) and *Blautia* (*P* < 0.01) and decreased the abundances of *Turicibacter* (*P* < 0.05), *Collinsella* (*P* < 0.01), and *Roseburia* (*P* < 0.05, Figure 4(c)).

### 3.3. Effects of JGXZ on Gut Permeability and Inflammatory Response in NAFLD Model Rats

**Figure 5:**
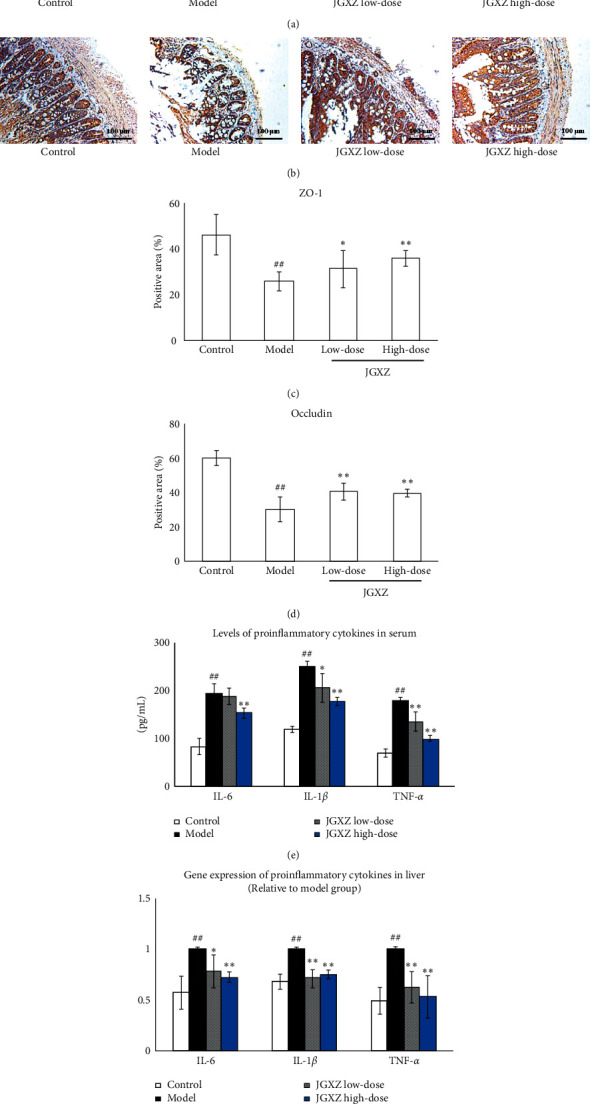
JGXZ treatment increased gut integrity and inhibited the inflammatory response in NAFLD model rats. (a–d) Immunostaining showed that the expression of ZO-1 (a, c) and occludin (b, d) in the colon was increased after JGXZ treatment. (e) The levels of IL-6, IL-1*β*, and TNF-*α* in the serum were decreased after JGXZ treatment. (f) The gene expressions of *Il6*, *Il1b*, and *Tnfa* in the liver were decreased after JGXZ treatment. Control, model, JGXZ low-dose, and JGXZ high-dose groups (*n* = 10 per group). ^#^*P* < 0.05 as compared to the control group; ^##^*P* < 0.01 as compared to the control group; ^∗^*P* < 0.05 as compared to the experimental model group; ^∗∗^*P* < 0.01 as compared to the experimental model group.

Immunostaining indicated that ZO-1 and occludin were expressed in all colonic epithelial cells. The expressions of ZO-1 and occludin were decreased in the model group compared with the control (*P* < 0.01; Figures 5(a)–5(d)). JGXZ treatment (8 and 16 g crude herb/kg) increased the expressions of ZO-1 (*P* < 0.05 and *P* < 0.01, respectively; Figures 5(a) and 5(c)) and occludin (*P* < 0.01; Figures 5(b), 5(d)). The serum levels of IL-6, IL-1*β*, and TNF-*α* were increased in NAFLD model rats compared with rats receiving standard chow (*P* < 0.01; Figure 5(e)). The serum levels of IL-1*β* and TNF-*α* were lower in the JGXZ low-dose group than those in the model group (*P* < 0.05 and *P* < 0.01, respectively; Figure 5(e)). High-dose JGXZ treatment decreased the serum levels of IL-6, IL-1*β*, and TNF-*α* in NAFLD model rats (*P* < 0.01; Figure 5(e)). Likewise, the gene expressions of *Il6*, *Il1b*, and *Tnfa* in the liver were upregulated in the model group compared with the control group (*P* < 0.01), whereas JGXZ treatment (8 and 16 g crude herb/kg) downregulated the gene expressions of *Il6* (*P* < 0.05 and *P* < 0.01, respectively), *Il1b* (*P* < 0.01), and *Tnfa* (*P* < 0.01; Figure 5(f)).

## 4. Discussion

In this study, we established a NAFLD rat model using HFD. Our results showed that the body weights and liver indices were increased in rats received HFD. Moreover, NAFLD model rats exhibited significant body weight gain, dyslipidemia, and hepatic steatosis, which are consistent with the pathological changes of NAFLD. In agreement with our previous study, JGXZ treatment (8 and 16 g crude herb/kg) showed remarkable therapeutic effects on NAFLD, manifesting as an improvement of body weight gain, liver index, dyslipidemia, and pathological changes in liver.

In addition, we investigated changes in gut microbiological composition using high-throughput sequencing. HFD has been shown previously to cause an increase in the alpha diversity of gut microbiota [20]. Likewise, our results showed a higher Shannon index in HFD-treated rats compared with the control group. JGXZ (16 g crude herb/kg) treatment reduced the alpha diversity of gut microbiota in NAFLD rats. PCoA analysis revealed significant distances between control and NAFLD model rats, indicating that the beta diversity of gut microbiota differed in HFD rats from rats received standard chow. According to the system clustering tree, the beta diversity of the gut microbiota between JGXZ (16 g crude herb/kg)-treated rats and control rats were more similar than that between the NAFLD model rats and control rats. Accumulating numbers of studies show that the *Firmicutes*-to-*Bacteroidetes* ratio (*F*-to-*B* ratio) is closely related to many metabolic diseases, including obesity, type 2 diabetes, and NAFLD [21–23]. Compared with the healthy subjects, NAFLD patients show a significant increase in the relative abundance of *Firmicutes* and a remarkable decrease in *Bacteroidetes*, resulting in an increase in the *F*-to-*B* ratio [24, 25]. Decreasing *F*-to-*B* ratios in the gut microbiota demonstrated a superior clinical outcome in NAFLD patients [26]. Animal studies also showed the increase in *F*-to-*B* ratios in the NAFLD model; *Lonicera caerulea* L. berry polyphenols could decrease the *F*-to-*B* ratio in NAFLD mice [27]. In agreement to these prior studies, our results indicated a remarkable increase in the *F*-to-*B* ratio in the model group, with JGXZ treatment decreasing this increased *F*-to-*B* ratio.

At the genus level, the abundances of *Lactobacillus* and *Blautia* were increased in JGXZ-treated rats. Previous studies have demonstrated that *Lactobacillus* and *Blautia* were decreased in NAFLD patients [28, 29]. Mulberry leaf fiber and polyphenols mixture have been used to induce weight loss in obese rats through increasing the abundance of *Lactobacillus* [30]. Similarly, Xie-Xin-Tang has been shown to increase the abundance of *Blautia* and improve the hyperglycemia, dyslipidemia, and inflammation in type 2 diabetes rats [31]. The relative abundances of *Turicibacter*, *Collinsella*, *Faecalibaculum*, and *Roseburia* were all increased in rats fed with HFD; JGXZ treatment decreased the gut abundances of *Turicibacter*, *Collinsella*, and *Roseburia*. Studies have shown that *Turicibacter* is positively correlated with metabolic phenotypes induced by HFD [32]. Ethanol extracts from marine *microalga Chlorella pyrenoidosa* have been shown to alleviate lipid metabolic disorders in HFD rats through decreasing the abundance of *Turicibacter* [33]. In addition, clinical studies have demonstrated that the abundance of *Collinsella* was significantly increased in atherosclerotic and nonalcoholic steatohepatitis (NASH) patients [34]. Additional correlation analysis indicated that *Collinsella* was positively related to the dysfunction of lipid metabolism in NASH patients [35]. *Collinsella* was also decreased in type 2 diabetic patients after they received a structured weight loss program [36]. Decreasing the abundances of *Turicibacter* and *Collinsella* might be the mechanism of JGXZ on dyslipidemia. Changes in dietary habits could be responsible for changes in *Faecalibaculum* abundance. For example, one study found that the abundance of *Faecalibaculum* was increased in mice received whole-egg powder [37]. As for the changes in *Roseburia*, the abundance of *Roseburia* has been shown to increase in aged mice. However, few studies have demonstrated the role of *Faecalibaculum* or *Roseburia* in NAFLD, and further study is required before conclusions on their importance can be drawn. Furthermore, accumulated studies have demonstrated that compounds and herbs such as curcumin [38], *Astragalus* membranaceus polysaccharide [39], nuciferine [40], and *Radix Paeoniae Rubra* [41] in JGXZ could regulate gut microbiota. Further studies could be carried out to identify the critical compounds of JGXZ with regulatory effects on gut microbiota.

As the dysfunction in gut microbiota can disrupt the tight junction of intestinal epithelial cells, triggering an increase in gut permeability and contributing to inflammatory response [42], we studied whether JGXZ could enhance the integrity of the intestinal mucosa barrier and ameliorate inflammatory response in NAFLD model rats. ZO-1 and occludin were examined as indicators as they are tight junction proteins expressed in the intestinal epithelial cells [43]. Occludin can influence the tight junction of intestinal epithelial cells through regulating macromolecule flux [44]. ZO-1, also called tight junction protein 1, is a cytoplasmic plaque protein that connects the transmembrane proteins to cytoskeleton [45, 46]. Decreases in ZO-1 and occludin expression in the gut indicate a reduction of intestinal epithelial cell integrity [47]. Accordingly, reduced levels of ZO-1 and occludin were observed in HFD-treated rats compared with control rats, whereas JGXZ treatment increased the protein expressions of both ZO-1 and occludin. Moreover, JGXZ treatment attenuated the inflammatory response in NAFLD model rats, manifesting as mild lobular liver inflammation and reduced expression of proinflammatory cytokines (IL-6, IL-1*β*, and TNF-*α*) in the serum and liver. Infiltration of inflammatory cells into the liver can induce excessive proinflammatory cytokine production, including IL-6, IL-1*β*, and TNF-*α*, and induce hepatocyte injury, contributing to NASH [48]. These proinflammatory cytokines can also disrupt lipid metabolism and cause dyslipidemia [49]. Previous studies have demonstrated that the colonization of *Lactobacillus rhamnosus* GG can prevent liver injury by improving the gut integrity and ameliorating liver inflammation in an alcoholic liver disease model [50]. *Lactobacillus rhamnosus* GG was also shown to improve intestinal barrier dysfunction in patients with irritable bowel syndrome [51]. *Blautia* has been reported to reduce the inflammatory response in obesity-related complications. *In vitro* studies have shown that *Blautia* can inhibit the production of proinflammatory cytokines in the LPS-induced activation of peripheral blood mononuclear cells [52]. Based on these previous research studies, we postulate that the effects of JGXZ on gut permeability and liver inflammation occur via affecting the abundances of *Lactobacillus* and *Blautia*. In addition, several studies have shown that the dysbiosis of gut microbiota could cause intestinal inflammation and further contribute to the inflammatory response in the liver [53]. Modulating the gut microbiota to alleviate intestinal inflammation could reduce liver inflammation in HFD-induced metabolic syndrome animal models [54]. The alternation of intestine-related inflammatory factors introduced by JGXZ treatment could be investigated in our future studies to demonstrate whether JGXZ has the potential to improve inflammatory response in the liver through modulating gut microbiota and alleviating intestinal inflammation.

## 5. Conclusion

In conclusion, our current study demonstrated that JGXZ could ameliorate NAFLD through modulating gut microbiota, decreasing gut permeability, and alleviating inflammatory response (sFigure1). Rats received high-dose JGXZ treatment exhibited superior therapeutic outcomes—more significant improvement in gut microbiota dysbiosis, lower grade of inflammation, and higher gut permeability—compared with low-dose JGXZ-treated rats, highlighting JGXZ's mechanisms and therapeutic potential against NAFLD in a dose-dependent manner.

## Figures and Tables

**Table 1:** Primer sequences used for the target rat genes.

Genes	Primer sequence (5′–3′)
*Actb*	Forward: TCTTCCAGCCTTCCTTCCTG
	Reverse: CACACAGAGTACTTGCGCTC

*Il6*	Forward: CTCATTCTGTCTCGAGCCCA
	Reverse: TGAAGTAGGGAAGGCAGTGG

*Il1b*	Forward: GGGATGATGACGACCTGCTA
	Reverse: TGTCGTTGCTTGTCTCTCCT

*Tnfa*	Forward: GAGCACGGAAAGCATGATCC
	Reverse: TAGACAGAAGAGCGTGGTGG

**Table 2:** Changes in serum ALT, AST, TG, and TC levels in NAFLD model rats.

Group	ALT (U/L)	AST (U/L)	TG (mmol/L)	TC (mmol/L)
Control	36.87 ± 8.61	81.33 ± 16.15	2.06 ± 0.49	4.46 ± 1.26
Model	77.02 ± 18.80^##^	166.82 41.02^##^	10.67 ± 2.07^##^	6.82 ± 1.51^##^
JGXZ low-dose	53.12 ± 21.33^*∗*^	145.19 ± 38.16	7.25 ± 2.60^*∗*^	6.05 ± 2.08
JGXZ high-dose	45.80 ± 9.97^∗∗^	129.81 ± 17.02^∗∗^	6.37 ± 1.57^∗∗^	4.69 ± 1.49^*∗*^

Control, model, JGXZ low-dose, and JGXZ high-dose groups (*n* = 10 per group). Data are presented as the mean ± SD.^##^*P* < 0.01 compared to the control group.^*∗*^*P* < 0.05 compared to the experimental model group.^∗∗^*P* < 0.01 compared to the experimental model group.

## Data Availability

The data sets used and/or analyzed during the current study are available from the corresponding author on reasonable request.

## Supplementary Materials

Supplementary Materials sFigure1. Graphical abstract. JGXZ could ameliorate NAFLD through modulating gut microbiota, decreasing gut permeability, and alleviating liver inflammation.
