# Supplementary material for: Jian-Gan-Xiao-Zhi Decoction Alleviates Inflammatory Response in Nonalcoholic Fatty Liver Disease Model Rats through Modulating Gut Microbiota
Source: Evid Based Complement Alternat Med. 2021 Mar 20;2021:5522755. doi: 10.1155/2021/5522755 (PMC8007356; doi:10.1155/2021/5522755)
Supplement: Supplementary Materials — sFigure1. Graphical abstract. JGXZ could ameliorate NAFLD through modulating gut microbiota, decreasing gut permeability, and alleviating liver inflammation. [file 5522755.f1.docx]

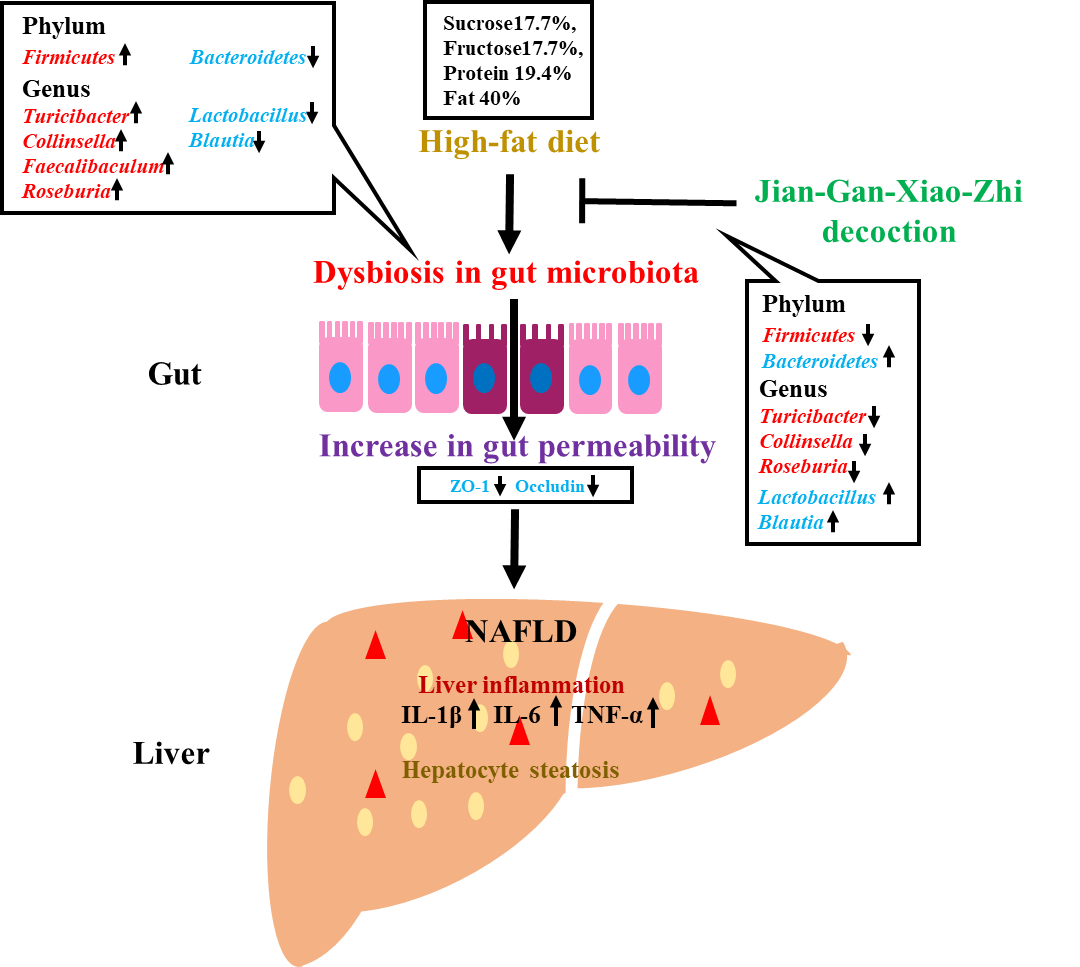


**sFigure1 Graphical abstract.**

JGXZ could ameliorate NAFLD through modulating gut microbiota, decreasing gut permeability, and alleviating liver inflammation.
